# A Study of the Effects of Oleuropein and Polydatin Association on Muscle and Bone Metabolism

**DOI:** 10.3390/biom15050628

**Published:** 2025-04-28

**Authors:** Maria Beatrice Morelli, Cristina Aguzzi, Riccardo Rascioni, Fiorenzo Mignini

**Affiliations:** 1 School of Pharmacy, University of Camerino, 62032 Camerino, Italy; mariabeatrice.morelli@unicam.it (M.B.M.); cristina.aguzzi@unicam.it (C.A.); 2 International Institute for Clinical Research and Analisys (IICRA srl), Spin Off University of Camerino, 63032 Camerino, Italy; riccardo.rascioni@arpacosmetics.it

**Keywords:** oleuropein, polydatin, human osteoblast, human myoblast, cell differentiation

## Abstract

Sarcopenia and osteoporosis are age-related musculoskeletal pathologies that often develop in parallel, and numerous studies support the concept of a bone–muscle unit, where deep interaction between the two tissues takes place. In Mediterranean areas, the lowest incidence of osteoporosis within Europe is observed, so the Mediterranean diet was suggested to play an important role. Consequently, in this study, oleuropein, a phenolic compound found in olive oil, and polydatin, another natural polyphenol found in the Mediterranean diet, were evaluated to determine their beneficial effects on bone and muscle metabolism. In human osteoblasts and skeletal muscle myoblasts, the effects were examined, and, after analyzing the cytotoxic effect to find non-toxic doses, the modulation of bone and muscle differentiation markers was evaluated at the gene and protein levels using PCR, Western blot, and immunohistochemistry. Interestingly, the compounds increased markers involved in osteoblast differentiation, such as osteocalcin, type I collagen, and dentin-sialo-phosphoprotein, as well as markers involved in myoblast differentiation, such as myogenic regulatory factors and creatine kinase. These effects were most noticeable when the compounds were administered together. These results suggest a beneficial role for oleuropein–polydatin association on bone and muscle tissue pathologies simultaneously.

## 1. Introduction

Sarcopenia and osteoporosis are progressive, age-related musculoskeletal disorders that frequently develop in parallel, significantly impacting mobility, independence, and overall health in older adults. Osteoporosis is defined by a decrease in bone mineral density and structural deterioration, leading to increased susceptibility to fractures, particularly in weight-bearing bones such as the hip, spine, and wrist. Sarcopenia, on the other hand, is characterized by a progressive loss of skeletal muscle mass, strength, and function, increasing the risk of falls and subsequent injuries. During growth and development, bone and muscle maintain a proportional relationship, where mechanical loading from muscle contractions stimulates bone formation and strength. This interdependence forms the foundation of the biomechanical interaction theory, which suggests that bone adapts to muscle forces to optimize structural integrity and function throughout life [1,2,3]. Disruptions in this relationship with aging contribute to both osteoporosis and sarcopenia, leading to a vicious cycle of musculoskeletal decline, frailty, and increased fracture risk.

Recent studies emphasize the importance of muscle–bone crosstalk, mediated not only by mechanical forces, but also by biochemical signaling pathways involving myokines and osteokines. These molecular interactions suggest that sarcopenia and osteoporosis are not merely coexisting conditions, but are functionally interconnected at both the biomechanical and cellular levels [4,5]. Understanding these complex interactions may provide new insights into targeted interventions, including resistance training, nutrition, and pharmacological therapies, aimed at simultaneously preserving bone and muscle health to reduce the burden of age-related musculoskeletal diseases.

Numerous studies support the concept of a bone–muscle unit, wherein a continuous crosstalk between these two tissues occurs. This communication is mediated by bioactive molecules, including myokines secreted by skeletal muscle, which influence bone metabolism, and osteokines released by osteoblasts and osteocytes, which, in turn, modulate muscle function [5,6,7]. While the mechanical and biochemical interactions between bone and muscle have been extensively studied, the endocrine relationship between these tissues remains less understood. However, it is now well established that skeletal muscle secretes myokines into the circulation in response to exercise, which exert systemic effects on multiple organs, including bone [8]. Recent discoveries have further expanded our understanding of bone-derived signaling molecules. Osteocalcin, an osteoblast-derived protein, has been shown to impact muscle metabolism and function [7,9,10,11]. In experimental models, exogenous osteocalcin administration has been demonstrated to enhance muscle mass and strength in aged mice, suggesting a direct regulatory role in muscle physiology [12,13]. These findings highlight the potential for bone-derived factors to influence muscle homeostasis, further reinforcing the concept of bone–muscle crosstalk as a dynamic and bidirectional process.

Currently, there are no FDA-approved pharmacological treatments for sarcopenia, leaving resistance training as the primary intervention to improve muscle strength and prevent falls. However, the efficacy of resistance training declines with age and in individuals affected by chronic diseases, necessitating alternative therapeutic strategies. While myostatin inhibitors are emerging as a potential pharmacological approach, additional drug development efforts are needed to maintain or restore a healthy musculoskeletal system. Given the strong interdependence of bone and muscle, the simultaneous targeting of both tissues represents a promising avenue for future therapeutic strategies. The development of dual-action drugs capable of improving both bone and muscle quality could have significant clinical implications, particularly in aging populations at risk for osteoporosis and sarcopenia.

Due to the multifactorial nature of these disorders, preventive strategies aimed at reducing the risk of osteoporosis and sarcopenia must be prioritized and implemented [14]. Although calcium and vitamin D supplementation are commonly recommended, recent evidence suggests that these measures alone may not be sufficient to counteract bone loss. Consequently, there has been growing interest in natural compounds with potential bone- and muscle-protective effects [15].

Epidemiological studies indicate that within Europe, the Mediterranean region exhibits the lowest incidence of osteoporosis, suggesting that dietary factors may play a crucial role in musculoskeletal health. Among these, the consumption of olive oil phenols, a hallmark of the Mediterranean diet, has been proposed as a key contributor to skeletal protection [16,17,18]. The Mediterranean diet is well-documented for its association with a reduced prevalence of cardiovascular diseases, neurodegenerative disorders, and certain cancers [19,20,21,22]. Oleuropein, hydroxytyrosol, and tyrosol—three major phenolic compounds in olive oil—are derived from the enzymatic hydrolysis of glycoside, a process that enhances their bioavailability [23,24]. Olive-derived polyphenols are primarily known for their antioxidant and anti-inflammatory properties [25]. In vitro studies suggest that oleuropein may promote osteoblastogenesis while inhibiting adipocyte differentiation, potentially favoring bone formation over fat accumulation within the bone marrow microenvironment [26,27]. Although preclinical studies indicate beneficial effects of oleuropein on bone mineral density and skeletal muscle mass, clinical trials evaluating its direct impact on the musculoskeletal system are still lacking [28,29,30,31].

Polydatin, also known as piceid, is another natural polyphenol predominantly isolated from the root and rhizome of Polygonum cuspidatum (Polygonaceae) [32]. It is also a common dietary component of the Mediterranean diet, which is associated with a broad spectrum of health benefits [33]. Since 2015, polydatin has been investigated for its potential protective effects against bone and joint disorders, with in vitro and in vivo studies suggesting its therapeutic relevance in humans [34]. Emerging evidence supports its role in mitigating osteoporosis, osteoarthritis, and rheumatoid arthritis (RA). The beneficial effects of polydatin are largely attributed to its pro-osteogenic, anti-inflammatory, antioxidant, apoptosis-modulating, and autophagy-regulating properties [35]. In vitro studies indicate that polydatin influences bone marrow stromal cell (BMSC) migration, differentiation, apoptosis, and oxidative stress responses [36]. Its anti-osteoporotic effects in vivo appear to be mediated by both enhanced bone formation and the inhibition of bone resorption. In osteoarthritis and RA, polydatin has been shown to suppress the secretion of inflammatory mediators implicated in cartilage and bone degradation [35].

In this study, we focused our research on the combined effects of a dry extract of olive leaves, standardized to 70% oleuropein, and pure polydatin on markers associated with bone and muscle metabolism. By investigating these natural compounds, we aim to explore their potential role in supporting musculoskeletal health and mitigating the progression of age-related bone and muscle loss.

## 2. Materials and Methods

### 2.1. Chemicals

Olive leaves extract titrated in 70% oleuropein and polydatin were purchased from INNBIOTEC Pharma (Arezzo, Italy) (Figure S1). Stock solution was prepared in DMSO and stored at −20 °C. The molarity values reported in this article for both compounds correspond to the concentrations of oleuropein and polydatin within the respective extracts used.

### 2.2. Human Cell Lines

Immortalized human osteoblast cell line CI-huOB (INS-CI-1005; InSCREENeX, Braunschweig, Germany) was maintained in huOB Maintenance Medium (INS-ME-1006; InSCREENeX). Osteogenic differentiation was induced by culturing huOBs in huOB Differentiation Medium (INS-ME-1007; InSCREENeX, Braunschweig, Germany). Immortalized Human Skeletal Muscle Myoblasts were also purchased (T0033; abm, Richmond, BC, Canada). Myoblast cultures were expanded in Prigrow III medium (TM003; Applied Biological Materials Inc. (abm), Richmond, BC, Canada) supplemented with fetal bovine serum (TM999; Applied Biological Materials Inc. (abm), Richmond, BC, Canada) to a final concentration of 10%, and Penicillin/Streptomycin (G255; Applied Biological Materials Inc. (abm), Richmond, BC, Canada) to a final concentration of 1% in ECM-coated culture flasks. Prigrow III medium supplemented with 2% horse serum (differentiation medium) was used to induce differentiation when cells reached confluence. Normal humidified 37 °C, 5% CO_2_ conditions were used for cell culture. The selection of these two cell lines was specifically driven by their ability to grow under proliferative and differentiated conditions, allowing for a comprehensive assessment of the effects of oleuropein and polydatin on bone and muscle cells.

### 2.3. MTT Assay

Three × 10^4^/mL huOBs and myoblasts were plated in 96-well plates and treated with different doses of oleuropein and or polydatin for 48 h. The samples were incubated for an additional 3 h with 0.8 mg/mL of 3-[4,5-dimethylthiazol-2-yl]-2,5 diphenyl tetrazolium bromide (MTT) (Sigma Aldrich, Milan, Italy). A microtiter plate spectrophotometer (BioTek Instruments, Winooski, VT, USA) was used to read the colored solutions after removing the medium from the wells and dissolving the formazan crystals in 100 mL of DMSO per well. Each experiment was replicated four times.

### 2.4. Alkaline Phosphatase Staining

During ossification, alkaline phosphatase activity was enhanced and could be detected by alkaline phosphatase staining. For staining, osteoblasts grown on 6-well plates were incubated for 60–120 s with 4% paraformaldehyde (PFA) and washed with (PBS + 0.05% Tween 20). Afterwards, the BCIP/NCT substrate solution (Sigma Aldrich, Milan, Italy) was added in each well for 20–30 min in the dark at an ambient temperature. The substrate 5-bromo-4chloro- 3indolyl phosphate (BCIP) can be converted by alkaline phosphatase to an indoxyl intermediate which further reacts with nitroblue tetrazolium (NBT) to an insoluble NBT diformazan product that is blue to purple in color. After incubation, the cells were analyzed with a bright field microscope.

### 2.5. Alizarin Red Staining

The presence of mineralization was confirmed by staining with alizarin red using an Osteogenesis Assay Kit (ECM815, Sigma Aldrich, Milan, Italy). Monolayer huOB cultures from day 21 differentiation were fixed in 4% PFA for 20 min at room temperature and stained with 2% alizarin red for 30 min at room temperature with gentle shaking. The extracellular calcium deposits on mineralized osteoblasts appear orange-red in color. For the colorimetric assay, 10% acetic acid was added in each well for 30 min with shaking and then heated to 85 °C for 10 min. Optical density (OD) was measured at 405 nm after neutralizing supernatant pH with 10% ammonium hydroxide.

### 2.6. Cell Cycle Analysis

During cell cycle analysis, ice-cold 70% ethanol fixed huOB and myoblast cells were treated with 100 μg/mL ribonuclease A solution for 30 min at 37 °C, stained with 20 μg/mL propidium iodide (PI, Sigma Aldrich, Milan, Italy) for 30 min at room temperature, and analyzed by flow cytometry using linear amplification.

### 2.7. Gene Expression Analysis

An RNeasy Mini Kit (Qiagen, Milan, Italy) was used to extract RNA from HuOB and myoblast cell lines. The A260/280 nm measurement was used to determine the concentration and purity of all RNA samples eluted in the appropriate buffer. The reverse transcription of 800 ng of RNA extracted was performed using the iScript kit (Bio-Rad, Hercules, CA, USA) according to the manufacturer’s instructions. One microliter of the resulting cDNA products, diluted to 1:10, was used as a template for digital droplet polymerase chain reaction (ddPCR).

Each sample was partitioned into 20,000 droplets, with targets and background (reference) cDNA randomly, but uniformly, distributed among the droplets. Up to 80 ng of cDNA, 2xddPCR supermix for probe and primers (Table S1), and deionized distilled water was used in the reactions. Bio-rad thermal cycler was used to run the emulsified PCR reactions. Incubation at 98 °C for 10 min was followed by 40 cycles of 95 °C for 15 s, 60 °C for 60 s, and 98 °C for 10 min. To assess the number of droplets positive for each target, the Bio-Rad QX200 droplet reader was used with QuantaSoft v1.4.0 software provided by Bio-Rad (Hercules, CA, USA). We used GAPDH and β-Actin as a reference for the amplification.

### 2.8. Western Blot

Lysate was obtained by lysing cells in a lysis buffer containing protease inhibitors (EuroClone, Milan, Italy). A Mini-PROTEAN Tetra Cell system (Bio-Rad, Hercules, CA, USA)) was used to separate proteins on 6.5–14% polyacrylamide gels. Bio-Rad’s Mini Trans-Blot Turbo RTA system was used to transfer protein from the gel to the nitrocellulose membrane. Blocking of non-specific binding sites was performed overnight at 4 °C with 5% low-fat dry milk and 2% bovine serum albumin (BSA). Membranes were incubated for 1 h at room temperature in primary Abs (anti-ALP, 1:100, Santa Cruz Biotechnology, Heidelberg, Germany; anti-OC, 1:200, Santa Cruz Biotechnology, Heidelberg, Germany; anti-COL1A, 1:200, Santa Cruz Biotechnology, Heidelberg, Germany; anti-DSPP, 1:200, Santa Cruz Biotechnology, Heidelberg, Germany; anti-Myf5, 1:300, Biorbyt Ltd. Cambridge, UK; anti-MyoG, 1:200, Santa Cruz Biotechnology, Heidelberg, Germany; anti-CKM, 1:300, Santa Cruz Biotechnology, Heidelberg, Germany; anti-VDR, 1:300, Santa Cruz Biotechnology, Heidelberg, Germany; anti-β-tubulin, 1:1000, Cell Signaling Technology, Milan, Italy; anti-GAPDH, 1:2000, Cell Signaling Technology, Milan, Italy), followed by incubation for 1 h at room temperature with HRP-conjugated anti-rabbit or anti-mouse secondary Abs. The detection was performed using the LiteAblot PLUS or Turbo kits (EuroClone, Milan, Italy), and densitometric analysis was carried out by ChemiDoc^TM^ XRS+ with Image Lab^TM^ Software version 6.1.0 (Bio-Rad, Hercules, CA, USA). For quantification, GAPDH or β-tubulin were used as loading controls. Every immunoblot figure shows one representative from three independent experiments.

### 2.9. Cell Death Analysis

For cell death analysis, huOB was treated with oleuropein and/or polydatin for 48 h and then incubated with 20 μg/mL PI for 20 min at 37 °C. Flow cytometry analysis was performed using BD CellQuest Pro software (version 5.1, BD Italia, Milan, Italy).

### 2.10. Statistical Analysis

The data are presented as the means and standard deviations (SDs) of three independent experiments. All obtained data underwent statistical analysis using the program GraphPad Prism^®^ 3.0 (GraphPad Software, San Diego, CA, USA). One-way ANOVA with Tukey’s multiple comparison test and Student’s *t*-test were used to determine statistical significance.

## 3. Results

### 3.1. Low Concentration of Oleuropein and Polydatin Stimulates Osteoblast Differentiation

**Figure 1:**
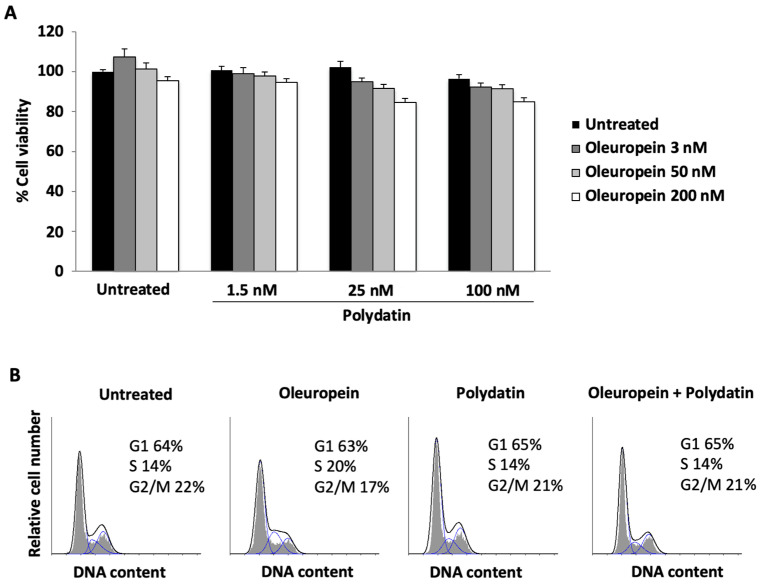
Effects of oleuropein and polydatin on the viability of huOB cells cultured under proliferative conditions. (**A**) Cell viability in huOB cells treated for 48 h with oleuropein 70% or polydatin, alone or in combination. The data are the means + SDs of three experiments. (**B**) Cell cycle analysis in huOB cells treated with oleuropein 3 nM and/or with polydatin 1.5 nM for 48 h. Numbers represent the percentages of cells in each cycle phase. The data are representative of three experiments.

First, we evaluated whether the growth of huOB was influenced by oleuropein under the proliferating condition. The results showed that doses from 3 to 50 nM have no effect on cell viability (Figure 1A). Instead, doses of 10 and 100 μM lead a statistically significant decrease in the cell viability percentage (Figure S2A), supported by an increase in PI incorporation, a sign of cell death (Figure S2B). Therefore, in the following experiments, we used an oleuropein concentration of 3 nM to exclude any potential cytotoxic effects. Also, polydatin does not show cytotoxic effects (Figure 1A), thus we have evaluated the effect of the combined use of the two compounds. None of the combinations tested appear to have an effect on cell viability, and this supported further studies (Figure 1A). To further confirm the ability of oleuropein and polydatin to alter cell viability, we performed a cell cycle analysis. The data showed that the 3 nM dose of oleuropein and the 1.5 nM dose of polydatin did not alter the cell cycle phase distribution (Figure 1B).

**Figure 2:**
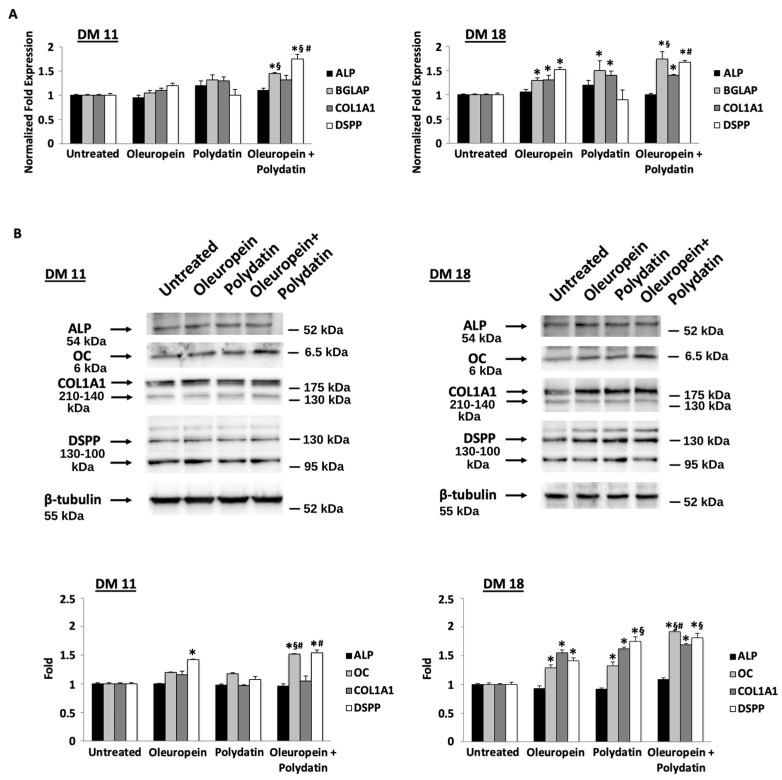
Oleuropein and polydatin coadministration stimulates osteoblast differentiation. (**A**) The relative ALP, BGLAP, COL1A, and DSPP mRNA expressions were evaluated by ddPCR in huOB treated with 3 nM oleuropein or 1.5 nM polydatin alone or in combination for 11 and 18 days in differentiation medium (DM). ALP, BGLAP, COL1A, and DSPP mRNA levels were normalized for β-actin expression. The data are expressed as the mean + SD. * *p* < 0.05 vs. untreated cells; ^§^ *p* < 0.05 as compared with oleuropein; and ^#^ *p* < 0.05 as compared with polydatin. (**B**) Cells are treated with oleuropein and polydatin as described above. Total lysates were separated on 6–14% SDS-PAGE and probed with anti-ALP, anti-OC, anti-COL1A, anti-DSPP, and anti-β-tubulin Abs. Blots are representative of one of three separate experiments. For statistical analysis, ALP, OC, COL1A, and DSPP densitometry values were normalized to β-tubulin levels. Folds represent changes with respect to untreated cells. The data are expressed as the mean + SD. * *p* < 0.05 vs. untreated cells; ^§^ *p* < 0.05 as compared with oleuropein; and ^#^ *p* < 0.05 as compared with polydatin. Original western blots can be found at Supplementary Materials.

**Figure 3:**
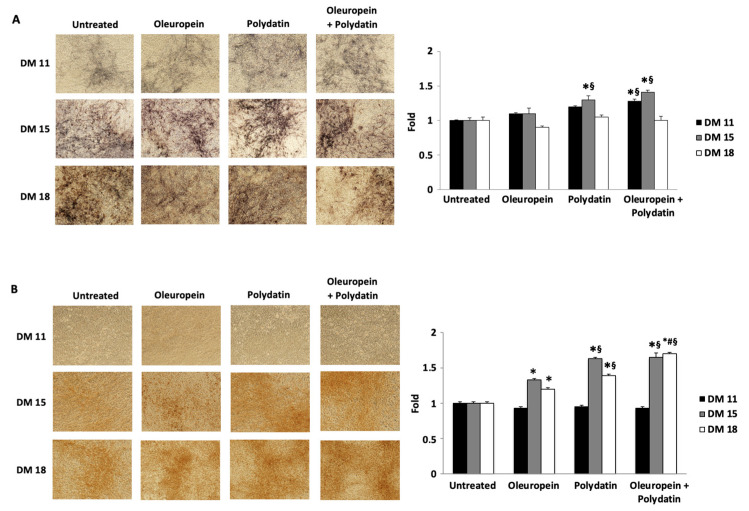
Oleuropein and polydatin effects on ALP activity and mineralization ability. Histochemical staining of ALP activity (**A**) and detection of mineralization visualized by Alizarin Red S staining (**B**) in monolayer cultures of huOB at 11, 15, and 18 days of differentiation, cultured in 3 nM oleuropein and/or 1.5 nM polydatin. Magnification 20×. Areas are representative of up to six fields of view collected from three independent experiments. Histograms represent quantitative data of ALP activity and quantitative data of Alizarin Red-S extraction expressed as fold values with respect to untreated cells. Values are obtained from three independent experiments and expressed as the mean + SD. * *p* < 0.05 as compared with control; ^§^ *p* < 0.05 as compared with oleuropein; and ^#^ *p* < 0.05 as compared with polydatin.

We evaluated the effects of the two compounds on osteoblast differentiation by determining the levels of alkaline phosphatase (ALP) expression, an early marker of osteoblast differentiation. At days 10 and 18 of differentiation, there were no differences between cells cultured in the presence of oleuropein or polydatin and control cells at the mRNA level, nor between their combinations (Figure 2A). The same results regarding ALP protein levels (Figure 2B) and ALP activity (Figure 3A) have been obtained by Western blot and ALP staining, respectively. However, an increase in ALP activity is noted in polydatin-treated cells at 15 days of differentiation, and this increase is more pronounced in the cells treated with the combination of the two compounds at 11 and 15 days of differentiation (Figure 3A).

Oleuropein’s effects on osteoblast differentiation were also determined by measuring the levels of mRNA and the protein expression of osteoblast differentiation and mineralization markers such as osteocalcin (OC) encoded by the bone gamma-carboxyglutamate protein (BGLAP) gene, type I collagen (COL1A1), and dentin-sialophosphoprotein (DSPP). Based on ddPCR, at day 18 of differentiation, cells cultured in oleuropein exhibited an increase in mRNA levels of these markers respect to control cells (Figure 2A). Polydatin influences positively BGLAP and COL1A1, whereas the oleuropein–polydatin combination induces an even greater increase in all three genes. Instead, Western blot analysis performed on oleuropein- and polydatin-treated huOB showed increased levels of DSPP at day 11, and OC, COL1A1, and DSPP at day 18 of differentiation (Figure 2B). Polydatin is able to induce OC increase only at 18 days of differentiation, and the combination confirms higher efficacy against OC and DSPP protein levels at 18 days of differentiation.

A mineralization staining test was then conducted to determine the effects of oleuropein and polydatin on late stages of osteoblast differentiation (Figure 3B). At day 11 of differentiation, there were no differences between the treated cells and the control. A statistically significant difference was observed between cells cultured with oleuropein and/or polydatin on day 15 and day 18 with respect to cells cultured without (Figure 3B).

### 3.2. Low Concentration of Oleuropein and Polydatin Stimulates Myoblast Differentiation

**Figure 4:**
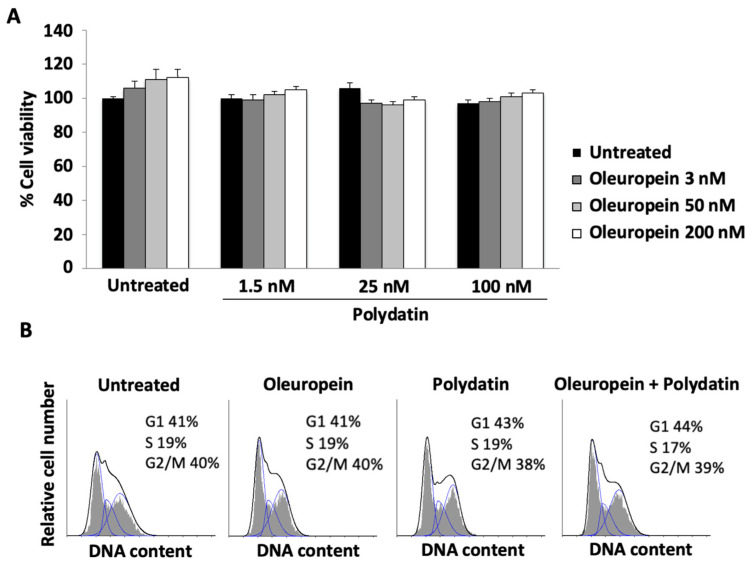
Effects of oleuropein and polydatin on the viability of myoblast cells cultured under proliferative conditions. (**A**) Cell viability in myoblast treated for 48 h with oleuropein and/or polydatin. Data are the mean + SD of three experiments. (**B**) Cell cycle analysis in myoblast treated with oleuropein 3 nM and/or with polydatin 1.5 nM. Numbers represent the percentages of cells in each cycle phase. Data are representative of three experiments.

Then, we determined whether the growth of human myoblast was influenced by oleuropein and polydatin. The results showed that there were no effects on cell viability when compounds are used alone or when they are used in combination (Figure 4A). Therefore, thanks to the results obtained in huOB, in the following experiments we used an oleuropein concentration of 3 nM and a polydatin concentration of 1.5 nM in myoblasts. To sustain the ability of the two compounds to alter cell viability, we performed a cell cycle analysis. The data showed that they do not alter the cell cycle phase distribution in myoblasts (Figure 4B).

**Figure 5:**
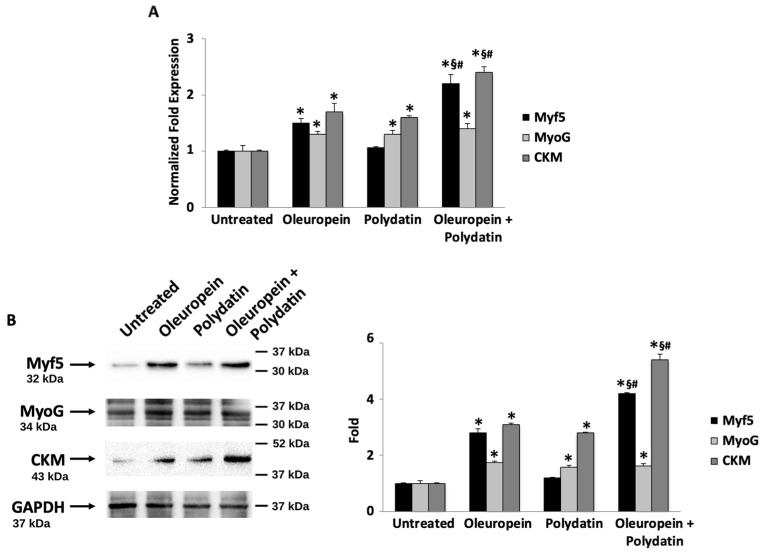
Oleuropein and polydatin combination stimulates myoblast differentiation. (**A**) The relative Myf5, MyoG, and CKM mRNA expressions were evaluated by ddPCR in myoblasts treated with 3 nM oleuropein and/or 1.5 nM polydatin for 3 days in differentiation medium. Myf5, MyoG, and CKM mRNA levels were normalized for β-actin expression. Data are expressed as mean + SD. * *p* < 0.01 as compared with control; ^§^ *p* < 0.01 as compared with oleuropein; and ^#^ *p* < 0.01 as compared with polydatin. (**B**) Cells were treated as described above, then total lysates were separated on 12–14% SDS-PAGE and probed with anti-Myf5, anti-MyoG, anti-CKM, and anti-GAPDH Abs. Blots are representative of one of three separate experiments. For statistical analysis, Myf5, MyoG, and CKM densitometry values were normalized to GAPDH levels. Folds represent changes with respect to untreated cells. Data are expressed as mean + SD. * *p* < 0.001 as compared with untreated cells; ^§^ *p* < 0.001 as compared with oleuropein; and ^#^ *p* < 0.001 as compared with polydatin. Original western blots can be found at Supplementary Materials.

In order to determine whether oleuropein and polydatin affect human myogenic differentiation, cells were cultivated in the differentiation medium for three days; then, myogenin (MyoG), a transcription factor essential to myogenic differentiation, myogenic factor 5 (Myf5), and creatin-kinase (CKM) expression levels were measured [37]. As shown in Figure 5A, the mRNA expression levels of Myf5, MyoG, and CKM were significantly higher with respect to control cells, indicating that myogenic differentiation was triggered by oleuropein. Polydatin is able to positively influence MyoG and CKM, whereas oleuropein–polydatin combination increases the differential markers even more (Figure 5A). The data were supported by immunoblot analysis supporting the fact that the combination is extremely effective in inducing myoblast differentiation (Figure 5B). Moreover, although all markers are upregulated, CKM exhibits a stronger response, potentially indicating a more pronounced effect on later stages of muscle differentiation.

### 3.3. Oleuropein–Polydatin Combination Stimulates Vitamin D3 Receptor Expression

**Figure 6:**
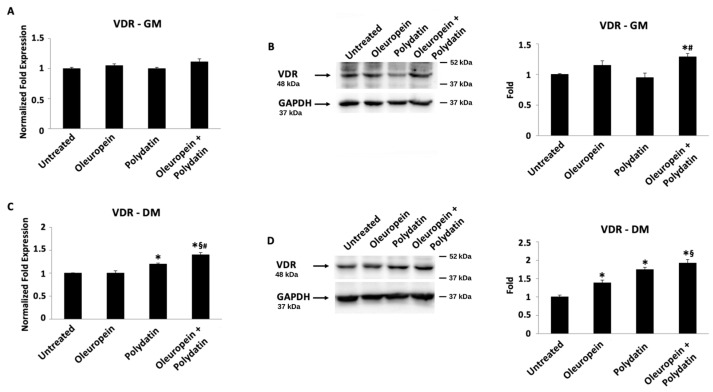
Oleuropein influences VDR expression. The relative VDR mRNA expression was evaluated by ddPCR in huOB treated with 3 nM oleuropein and/or 1.5 nM polydatin in growth medium (**A**) and for 18 days in differentiation medium (**C**). VDR mRNA levels were normalized for β-actin expression. Data are expressed as mean ± SD. * *p* < 0.05 vs. untreated cells; ^§^ *p* < 0.05 vs. oleuropein-treated cells; and ^#^ *p* < 0.05 vs. polydatin-treated cells. Cells treated with 3 nM oleuropein and/or 1.5 nM polydatin in growth medium (**B**) and for 18 days in differentiation medium (**D**) were analyzed by Western blot. Total lysates were separated on 9% SDS-PAGE and probed with anti-VDR and anti-GAPDH Abs. Blots are representative of one of three separate experiments. For statistical analysis, VDR densitometry values were normalized to GAPDH levels. Folds represent changes respect to untreated cells. Data are expressed as mean + SD. * *p* < 0.05 vs. untreated cells; ^§^ *p* < 0.05 vs. oleuropein-treated cells; and ^#^ *p* < 0.05 vs. polydatin-treated cells. Original western blots can be found at Supplementary Materials.

**Figure 7:**
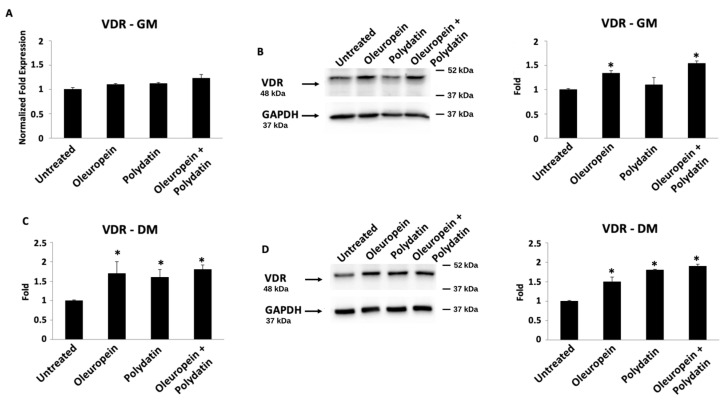
Oleuropein influences VDR expression. Relative VDR mRNA expression was evaluated by ddPCR in myoblasts treated with 3 nM oleuropein and/or 1.5 nM polydatin in growth medium (**A**) and for 3 days in differentiation medium (**C**). VDR mRNA levels were normalized for β-actin expression. Data are expressed as mean + SD. * *p* < 0.05 vs. untreated cells. Cells treated with 3 nM oleuropein and/or 1.5 nM polydatin in growth medium (**B**) and for 3 days in differentiation medium (**D**) were analyzed by Western blot. Total lysates were separated on 9% SDS-PAGE and probed with anti-VDR and anti-GAPDH Abs. Blots are representative of one of three separate experiments. For statistical analysis, VDR densitometry values were normalized to GAPDH levels. Folds represent changes with respect to untreated cells. Data are expressed as mean ± SD. * *p* < 0.05 vs. untreated cells. Original western blots can be found at Supplementary Materials.

Finally, the gene expression of the vitamin D3 receptor (VDR) was analyzed by ddPCR (Figure 6A,C and Figure 7A,C) and Western blot (Figure 6B,D and Figure 7B,D) techniques, both in huOB and in myoblasts. At mRNA levels, only polydatin and the combination are able to influence VDR transcription both in huOB and in myoblasts grown in the differentiation medium, compared to control cells (Figure 6C and Figure 7C). Instead, Western blot analysis showed about a 1.3-fold increase in VDR protein expression in huOB cells in the proliferative medium treated with the combination with respect to the control (Figure 6B). In the differentiative medium, huOB treated with oleuropein and the combination evidenced an increase in VDR protein expression (Figure 6D). Moreover, even though there are no changes in VDR mRNA expression in myoblasts cultured in the proliferative medium, its protein expression is improved after treatment with oleuropein or the combination (Figure 7B). Finally, in the differentiation medium, VDR showed the highest protein increase in myoblasts treated with oleuropein and polydatin alone or in combination (Figure 7D).

## 4. Discussion

Several studies have highlighted a strong association between sarcopenia and osteoporosis [38,39,40,41]. The physiological interaction between muscle and bone is increasingly recognized as a critical factor in preventing musculoskeletal diseases and disability in the elderly. In particular, Sjoblom et al. reported that women with sarcopenia have more than twice the risk of fractures and falls compared to those without the condition [42].

Despite the growing awareness of the detrimental impact of sarcopenia, there are currently no EMA- or FDA-approved pharmacological treatments for the disease. While myostatin inhibitors are emerging as potential therapeutic candidates, their clinical application remains limited. Therefore, there is an urgent need for novel pharmacological approaches aimed at preserving or restoring muscle mass and function. Developing effective interventions to target both muscle and bone simultaneously could represent a significant advancement in mitigating the burden of age-related musculoskeletal disorders. Some natural compounds are emerging as promising candidates for musculoskeletal health [43,44]. For instance, soy isoflavones have been shown to mitigate menopause-induced osteoporotic bone loss by reducing bone resorption and enhancing bone formation [45,46]. In parallel, they exhibit beneficial effects on skeletal muscle, particularly in response to muscle damage, high-intensity or high-speed exercise, and repeated bouts of strenuous physical activity [47]. These findings highlight their potential as multifaceted agents in the prevention and management of musculoskeletal decline. However, clinical trials are necessary to confirm their safety and efficacy in humans. Moreover, the inherent complexity of natural products—including variability in composition, bioavailability, and metabolism—can pose significant challenges in standardizing dosages and developing consistent formulations suitable for therapeutic use. Addressing these issues through further research will be essential to advance their translational potential and integration into clinical practice.

In this paper, we started to study the effects of olive leave extract titrated in oleuropein 70% and pure polydatin on human osteoblast and myoblast cells. By using these two models, we were able to study the potential dual role of these compounds in regulating both cell proliferation and differentiation, which is crucial for understanding their relevance in musculoskeletal health and their potential therapeutic applications in conditions such as osteoporosis and sarcopenia. In a relatively recent study, oleuropein was used at much higher concentrations (100 mM and 1 mM) than we used [26]; however, our analyses at 10^−6^ M doses already showed effects on cell culture. At 10 and 100 μM, the viability percentage decreased statistically significantly and PI incorporation increased, indicating cell death. Using this concentration, the authors were able to increase osteoblastogenesis and suppress adipogenesis in human bone marrow cultures. The same authors report that oleuropein-mediated gene modulation resulted in osteoblasts depositing more minerals in the presence of 10^−5^ or 10^−6^ M concentrations of the compound. Osteoblast differentiation, both in vitro and in vivo, occurs in three distinct stages: cell proliferation, matrix maturation, and matrix mineralization. In vitro, the processes of matrix maturation and mineralization are typically promoted by allowing the cells to reach full confluency and supplementing the culture medium with specific osteogenic factors that support bone formation. In this study, the data regarding both the gene expression and the production of the corresponding protein were obtained with low oleuropein and polydatin doses, which do not affect the proliferation of both osteoblasts and myoblasts. In fact, our data show that low concentrations of oleuropein and polydatin can stimulate osteoblast differentiation. Differentiated osteoblasts secrete an extracellular matrix rich in type I collagen, which undergoes calcification during the later stages of differentiation [48]; ALP is considered to be a marker of osteoblast differentiation, involved in the early stages of the mineralization process [49,50,51]. Our results demonstrated that oleuropein and polydatin promote ALP activity and calcium deposition in osteoblasts during late phases of differentiation.

Moreover, osteoblast maturation and mineralization are accompanied by a significant increase in the expression of the osteoblast-associated protein osteocalcin during the differentiation process [52]. Osteocalcin is widely recognized as a biochemical marker of osteogenesis, reflecting both the number and activity of osteoblasts [53]. In our model, the observed increase in osteocalcin levels aligns with findings from several preclinical and clinical studies investigating the effects of olive extract. Notably, one intervention trial involving 127 elderly men over a two-year period demonstrated that adherence to a Mediterranean diet enriched with olive oil was associated with increased plasma osteocalcin levels [19,20]. These results support further research to confirm the potential protective effects of polydatin and olive polyphenol extract on bone metabolism.

Beyond its role in bone formation, osteocalcin may also exert beneficial effects on skeletal muscle. Osteocalcin interacts with the G protein-coupled receptor family C group 6 member A (GPRC6A) in skeletal muscle, promoting glucose and fatty acid uptake and metabolism during exercise [54]. In parallel, osteocalcin stimulates muscle-derived interleukin-6 (IL-6) secretion, a cytokine that plays a key role in modulating the release of undercarboxylated osteocalcin (ucOC). This undercarboxylated form of osteocalcin has been implicated in metabolic regulation, as it enhances hepatic glucose production and stimulates the mobilization of fatty acids from adipose tissue [55,56].

During aerobic exercise, circulating levels of ucOC have been shown to double at the point when insulin reaches its lowest concentration, highlighting its role in metabolic adaptation. Furthermore, in aging models, osteocalcin has been found to be both necessary and sufficient for the maintenance of muscle mass. These findings suggest that osteocalcin plays a crucial role in the body’s adaptation to physical activity and contributes to the preservation of skeletal muscle mass, particularly in aging individuals [12,57]. Thus, through the increased osteocalcin production, oleuropein and polydatin can also exert beneficial multi-organ effects. Among analyzed markers, DSPP is an extracellular matrix, a prototypical dentin, and a bone-specific gene [58]. RUNX2 has been shown to differentially regulate DSPP promoter activity during the differentiation of odontoblasts [59]. At the molecular level, the DSPP gene undergoes multiple post-transcriptional modifications, resulting in the production of two major proteins: dentin sialoprotein (DSP) and phosphophoryn (PP) [60]. The precise functional significance of alternative splicing and the full role of DSPP remain unclear. Notably, DSPP expression is absent in mineralized dentin and odontoblasts. DSP is believed to play a crucial role in the early stages of dentin mineralization, while DPP is primarily involved in the maturation phase of dentin formation [61]. Thus, our results suggested that oleuropein and polydatin, increasing the expression of DSPP, could be able to promote the formation of reparative dentin, and could improve the differentiation of odontoblasts.

Moving on to the effect at the muscular level, low doses of oleuropein and polydatin do not affect the proliferative capacity of myoblasts here either, but manage to modulate their differentiation. Myogenic regulatory factors (MRFs) are essential regulators of myogenesis, functioning as a coordinated transcriptional cascade. MRFs belong to the basic Helix–Loop–Helix (bHLH) family of transcription factors and play a crucial role in both prenatal and postnatal muscle development. This family consists of four key members—Myf5, MyoD, MyoG, and MRF4—which are fundamental in guiding satellite cell commitment to the skeletal muscle lineage and promoting differentiation during muscle regeneration [62]. Our data evidenced that low concentrations of oleuropein and polydatin stimulate a significant increase in mRNA expression levels and protein production of Myf5, MyoG, and CKM compared to the control, a sign of myoblast differentiation. Oleuropein and polydatin may play a significant role in maintaining mature skeletal muscle integrity and, more importantly, in regulating satellite cell function to promote muscle regeneration. These polyphenols contribute to linking the genetic control of myogenesis by influencing key molecular pathways involved in muscle repair and growth. Specifically, they modulate the temporal and spatial expression patterns of myogenic regulatory factors, as well as their biochemical properties and hierarchical activation, primarily by regulating Myf5 and MyoG expression. Among these factors, MyoG plays a crucial role in facilitating the fusion of activated satellite cells, either with pre-existing muscle fibers or with each other, to form new myofibers. This process is essential for the regeneration of damaged skeletal muscle and the restoration of functional muscle tissue. By influencing these mechanisms, oleuropein and polydatin may enhance muscle repair efficiency, potentially counteracting muscle degeneration associated with aging or pathological conditions. Thus, supplementation with oleuropein and polydatin can contribute to the “programmability” of skeletal muscle. The degree of myoblast differentiation was also evaluated as a function of CKM expression. The amount of CKM is increased in the cells treated with oleuropein and polydatin following the formation of myotubes. We acknowledge that the current findings related to the differential modulation of CKM and MyoG represent preliminary observations that warrant further investigation. Due to the exploratory nature of this study, our focus was to provide initial evidence supporting the involvement of these markers in the analyzed context. Future studies will be designed to dissect these molecular dynamics more thoroughly, through time-course analyses and additional mechanistic approaches. These investigations will be instrumental in clarifying the specific pathways involved and in strengthening the biological relevance of our current findings.

Finally, we observed for the first time that oleuropein and its coadministration with polydatin increase VDR protein expression in osteoblasts, especially under differentiative conditions. The two polyphenols also stimulate VDR expression in myoblasts, both during the proliferative phase and during differentiation. In particular, these data suggest that oleuropein and polydatin act on VDR, especially at protein levels. Presumably, these compounds could increase the half-life of VDR by protecting it from proteasomal degradation, as demonstrated in human CD4+ T cells, depending on 1,25-Dihydroxyvitamin D3 [63]. These findings are particularly significant, as the vitamin D receptor (VDR) is not only a crucial regulator of bone mass in osteoblasts [64], but its deficiency in myoblasts has also been shown to impair muscle cell contractility [65]. Moreover, the number of VDRs declines with age, a factor that has been implicated in the progressive reduction in muscle strength observed in aging individuals [66,67]. Given these insights, the administration of oleuropein and polydatin in the elderly holds great promise, as they have been shown to enhance VDR expression. This is particularly relevant for individuals who have limited sun exposure and struggle to consume sufficient dietary polyphenols, both of which contribute to suboptimal vitamin D signaling. Therefore, supplementation with oleuropein and polydatin emerges as a potential strategy for preventing and managing sarcopenia by supporting muscle function and bone health. Beyond their direct effects on muscle and bone, VDR activation plays a central role in facilitating skeletal-muscle crosstalk and maintaining inter-organ communication. The dysregulation of this intricate network is a key contributor to movement disorders and musculoskeletal decline. Our findings suggest that targeting VDR upregulation through oleuropein and polydatin supplementation may offer a novel approach to counteract the progressive deterioration of this inter-organ system, thereby preserving neuromuscular function. These findings, although based on moderate fold changes, were obtained using relatively low concentrations of natural compounds, supporting the notion that the oleuropein/polydatin combination exerts a biologically meaningful activity. This indicates a promising pharmacological potential, particularly under physiological or stress-related conditions. To better characterize this potential, further studies are currently underway to investigate a broader dose–response range. These future investigations will not only help validate the observed effects, but also lay the groundwork for potential clinical applications of this natural compound combination.

In conclusion, given the strong bidirectional interaction between muscle and bone, oleuropein and polydatin may promote a beneficial synergy between these two tissues. This synergy could translate into tangible improvements in musculoskeletal health, enhancing quality of life not only in postmenopausal women and the elderly, but also in individuals affected by chronic pathological conditions associated with muscle and bone degeneration. Beyond osteoporosis and sarcopenia, these compounds may hold therapeutic potential in other musculoskeletal disorders, such as osteoarthritis, rheumatoid arthritis, and muscular dystrophies, where inflammation, oxidative stress, and impaired tissue regeneration are key pathological features. Furthermore, their effects on cellular metabolism and differentiation suggest possible applications in metabolic bone diseases, such as osteogenesis imperfecta, and in conditions marked by impaired muscle regeneration, including cachexia and neuromuscular disorders.

The results of this study provide a foundation for the development of additional experimental models using a similar approach, aimed at exploring the effects of these and other bioactive compounds in different cellular systems and pathological contexts. Future research could be extended to primary cells, 3D culture models, or in vivo studies to further elucidate the mechanisms underlying the musculoskeletal benefits of oleuropein and polydatin, with the potential to support novel therapeutic strategies for a wide range of degenerative conditions.

## 5. Conclusions

In summary, our findings suggest that the oleuropein/polydatin combination exerts biologically relevant effects on muscle and bone cells, even at low concentrations. This supports its potential as a pharmacologically active natural compound duo. Although fold changes were moderate, the consistent modulation of key differentiation markers highlights its promise in promoting musculoskeletal health. Given the close interplay between muscle and bone, these compounds could be valuable in addressing age-related or degenerative conditions. Overall, the results provide a strong rationale for the translational development of this combination towards future therapeutic strategies.

## 6. Patents

On the basis of this work, a patent was requested and obtained (Owner IICRA srl. Patent no.102019000002271).

## Data Availability

The original contributions presented in this study are included in the article/Supplementary Materials. Further inquiries can be directed to the corresponding author(s).

## Supplementary Materials

The following supporting information can be downloaded at https://www.mdpi.com/article/10.3390/biom15050628/s1. Figure S1: Oleuropein and polydatin structures. Figure S2: Oleuropein effects on huOB cell viability. Table S1: List of primers used in ddPCR assay. File S1: Original western blots figures.
