# Supplementary material for: A Study of the Effects of Oleuropein and Polydatin Association on Muscle and Bone Metabolism
Source: Biomolecules. 2025 Apr 28;15(5):628. doi: 10.3390/biom15050628 (PMC12109345; doi:10.3390/biom15050628)
Supplement: Supplementary file 1 [file biomolecules-15-00628-s001.zip › biomolecules-3505794-File S1.original-images.pdf]

Figure 2

B

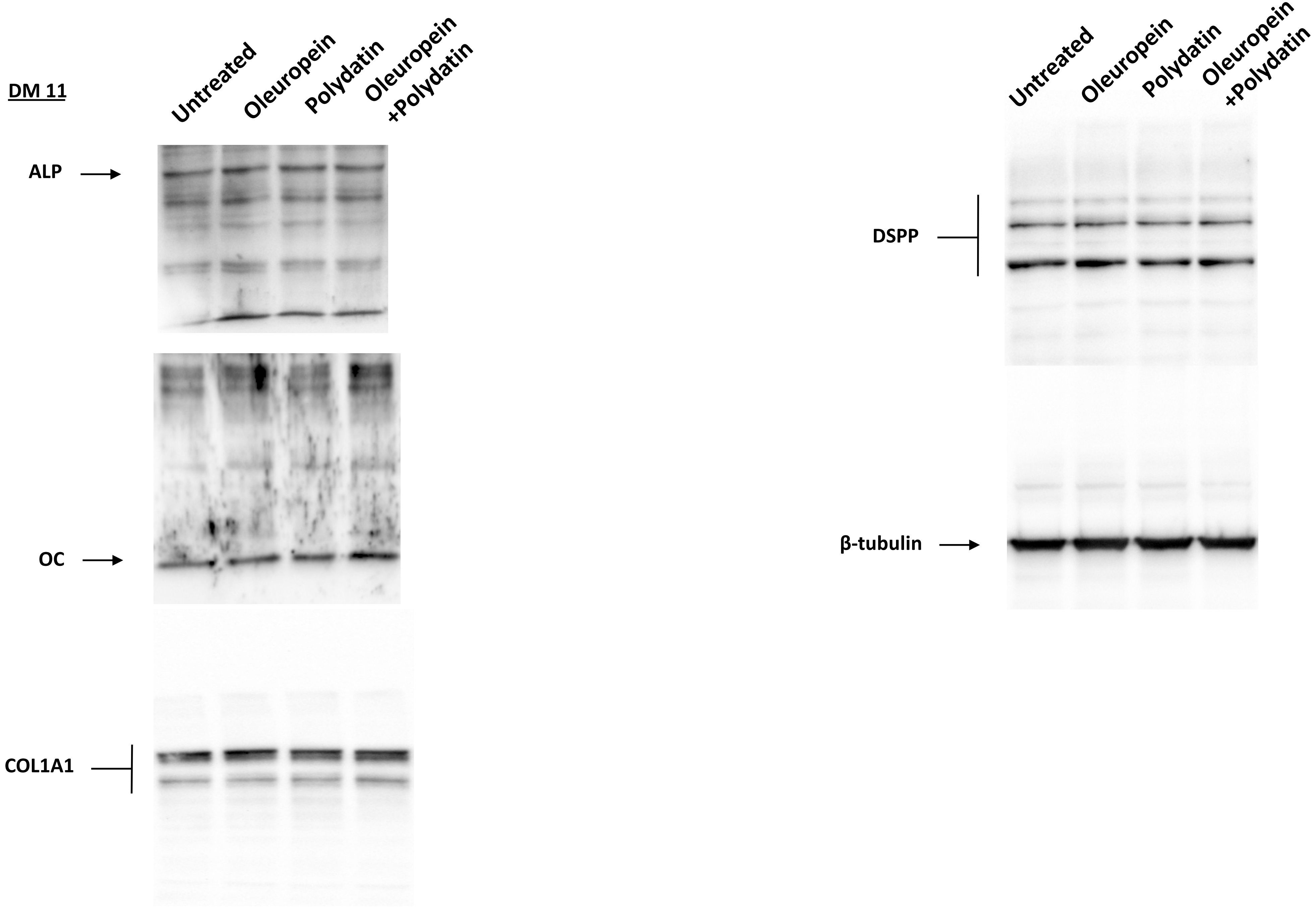
DM 11
Untreated
Oleuropein
Polydatin
Oleuropein +Polydatin
ALP
OC
COL1A1
Untreated
Oleuropein
Polydatin
Oleuropein +Polydatin
DSPP
β-tubulin

Figure 2

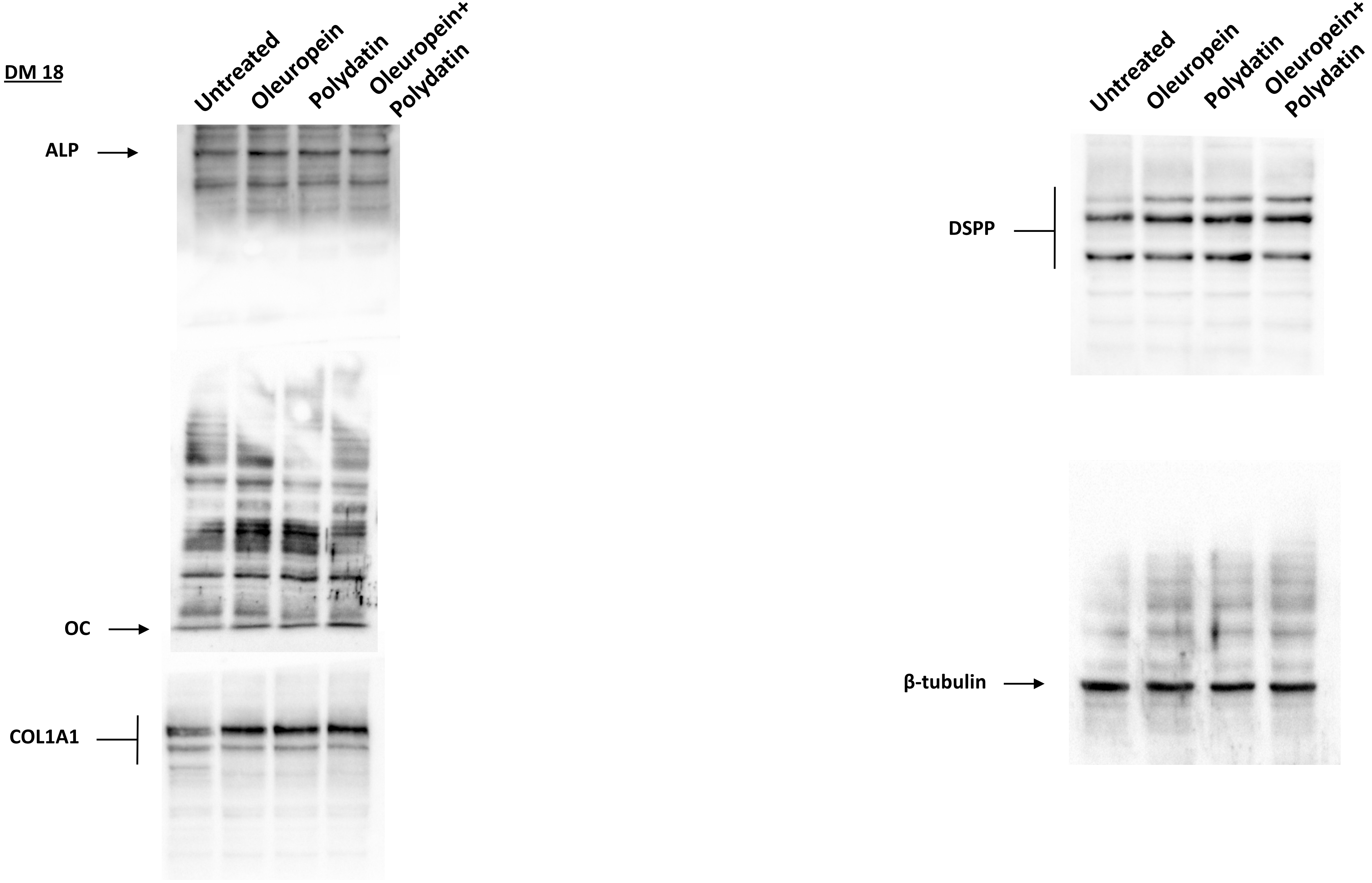
DM 18
Untreated
Oleuropein
Polydatin
Oleuropein+ Polydatin
ALP
OC
COL1A1
Untreated
Oleuropein
Polydatin
Oleuropein+ Polydatin
DSPP
β-tubulin

Figure 5

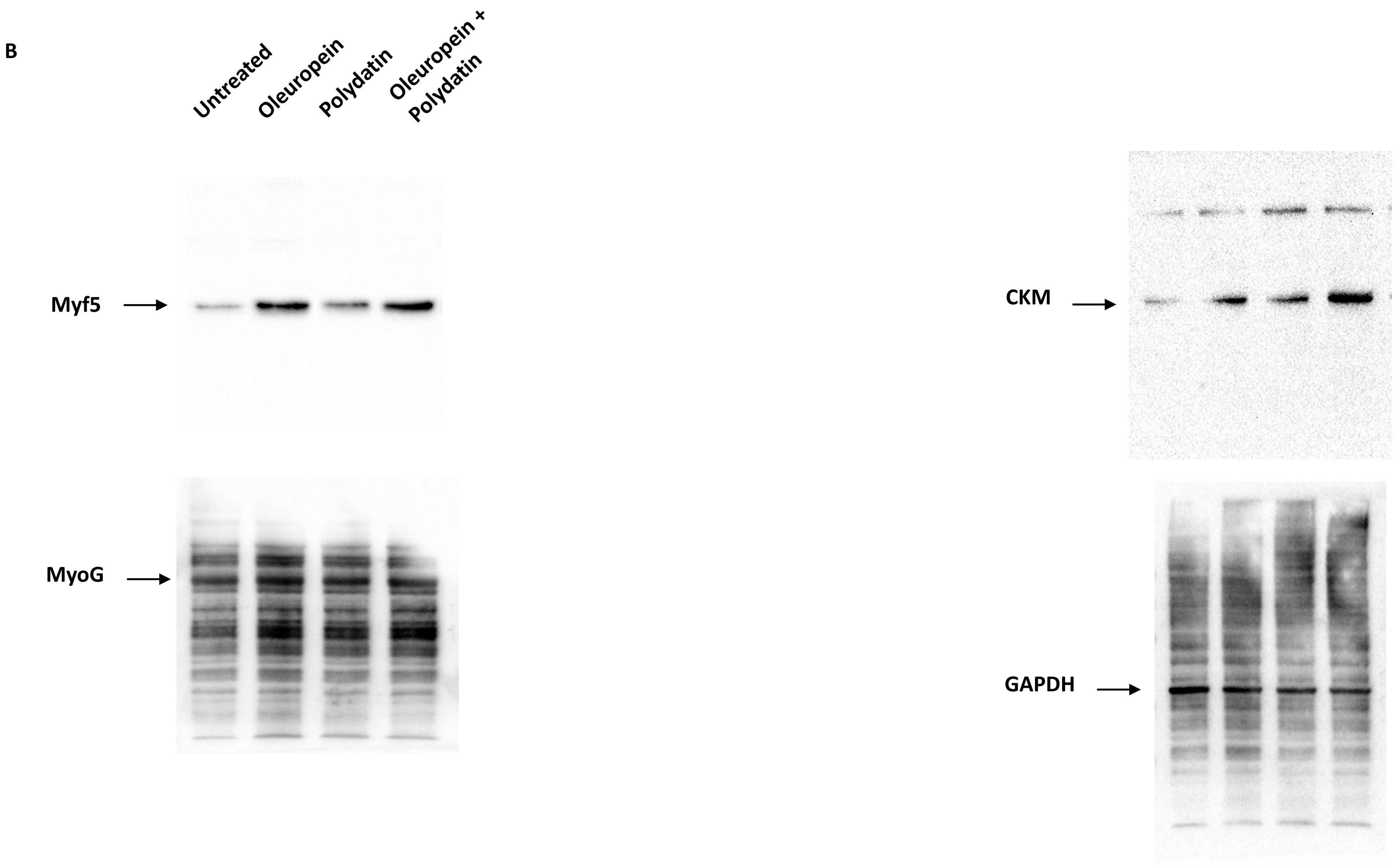
B
Untreated
Oleuropein
Polydatin
Oleuropein +
Polydatin
Myf5
MyoG
CKM
GAPDH

Figure 6

B

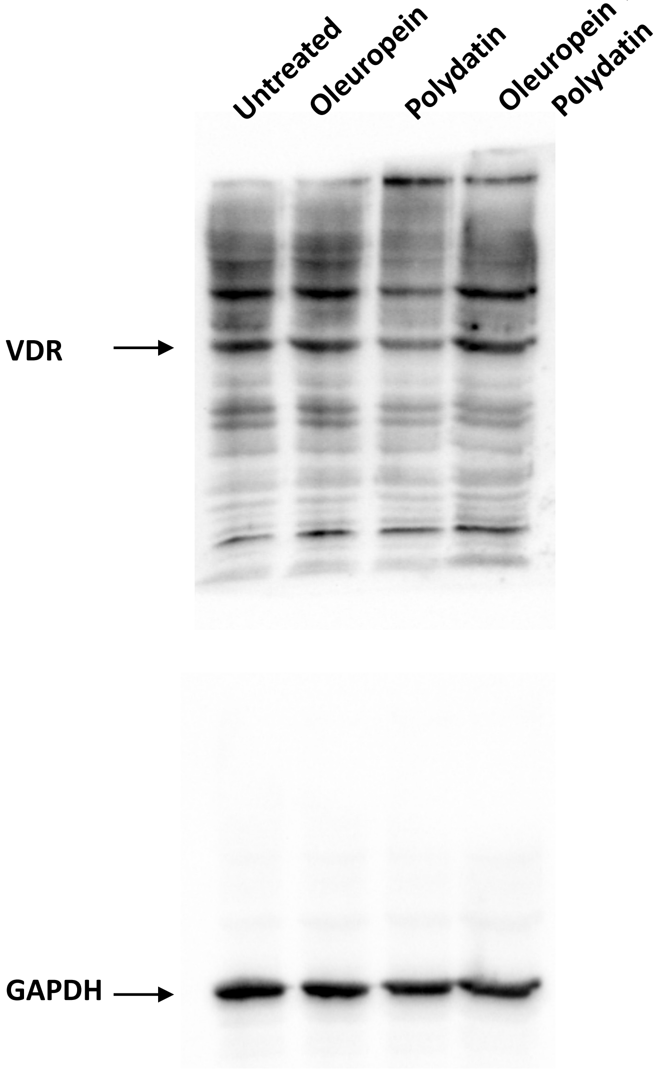

Untreated
Oleuropein
Polydatin
Oleuropein + Polydatin
VDR
GAPDH

D

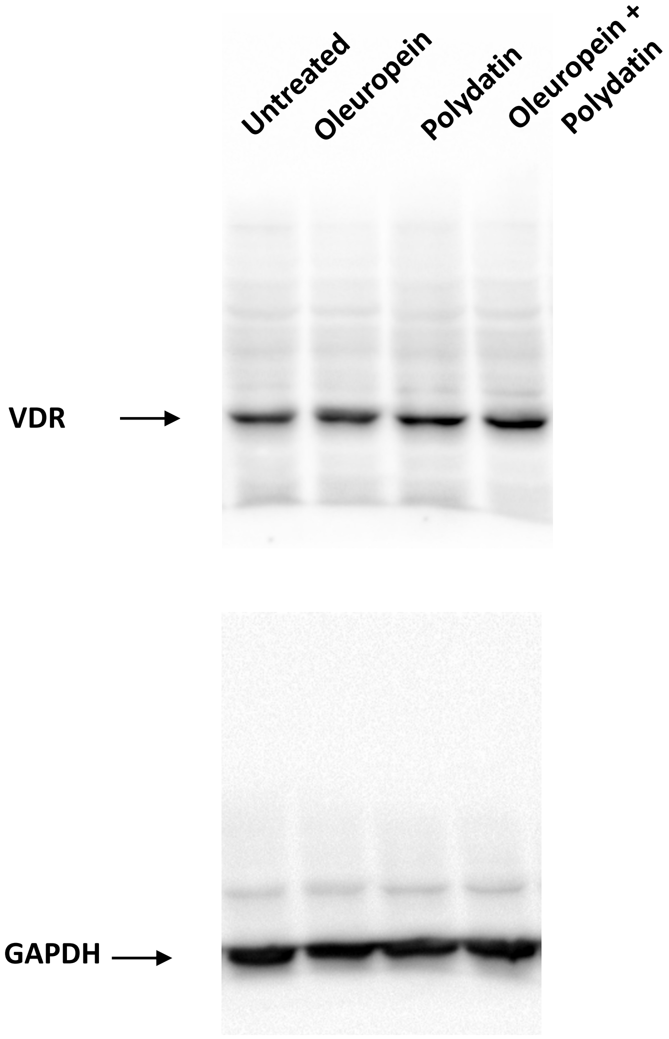

Untreated
Oleuropein
Polydatin
Oleuropein + Polydatin
VDR
GAPDH

Figure 7

B

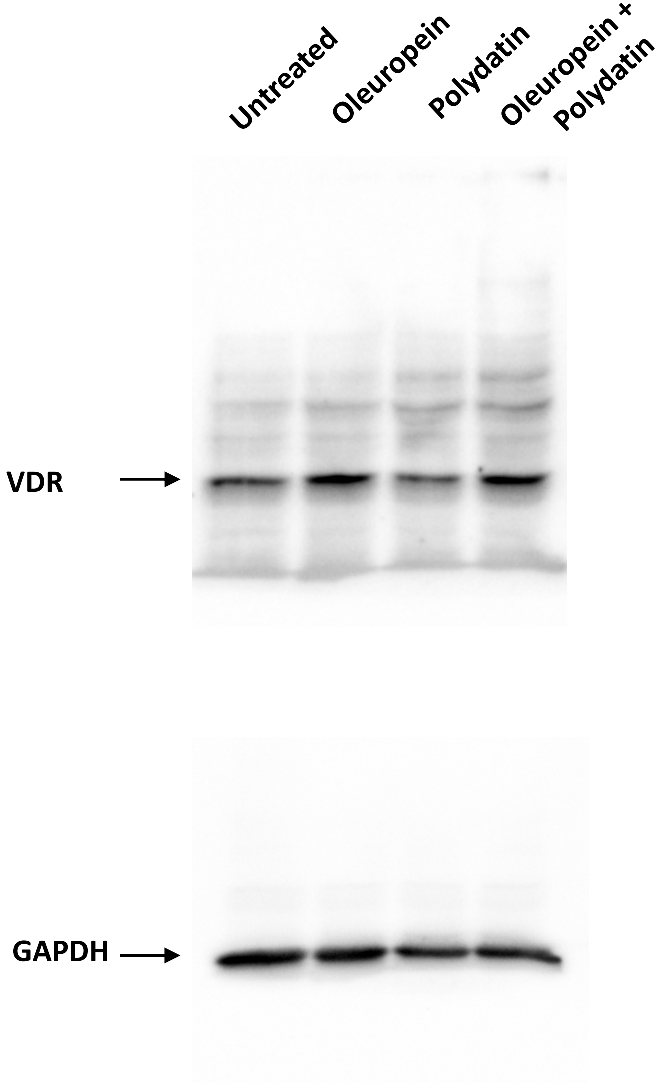

Untreated
Oleuropein
Polydatin
Oleuropein + Polydatin
VDR
GAPDH

D

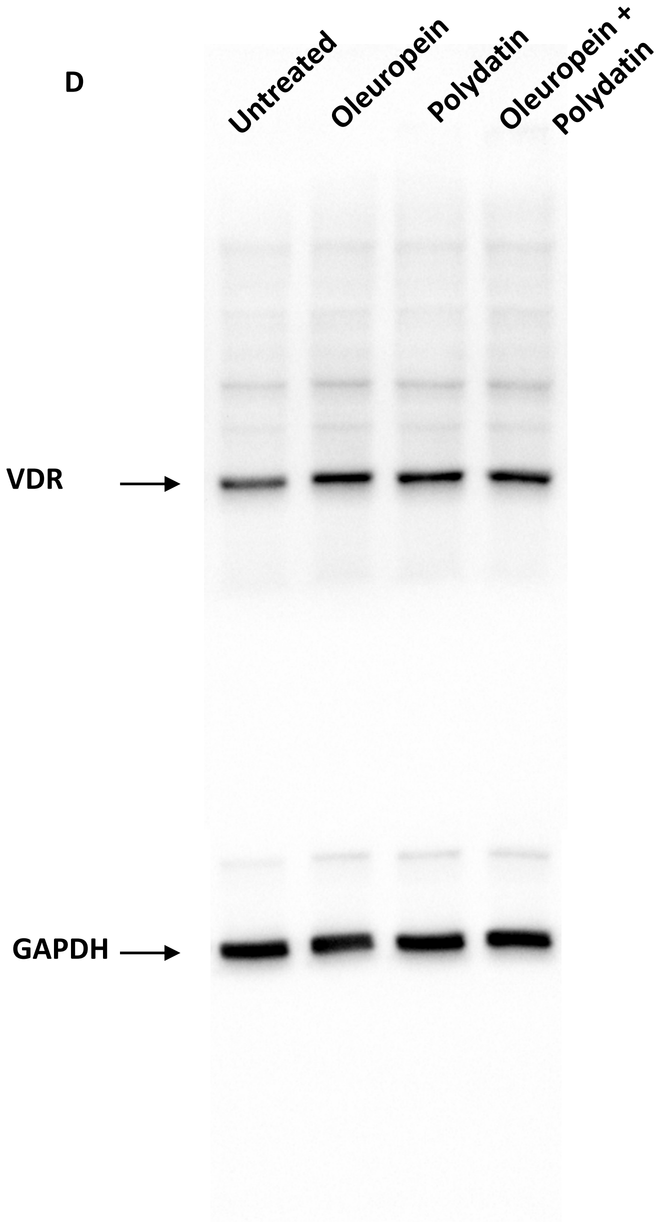

Untreated
Oleuropein
Polydatin
Oleuropein + Polydatin
VDR
GAPDH
