# Supplementary material for: A Study of the Effects of Oleuropein and Polydatin Association on Muscle and Bone Metabolism
Source: Biomolecules. 2025 Apr 28;15(5):628. doi: 10.3390/biom15050628 (PMC12109345; doi:10.3390/biom15050628)
Supplement: Supplementary file 1 [file biomolecules-15-00628-s001.zip › biomolecules-3505794-supplementary.pdf]

Supplementary Materials

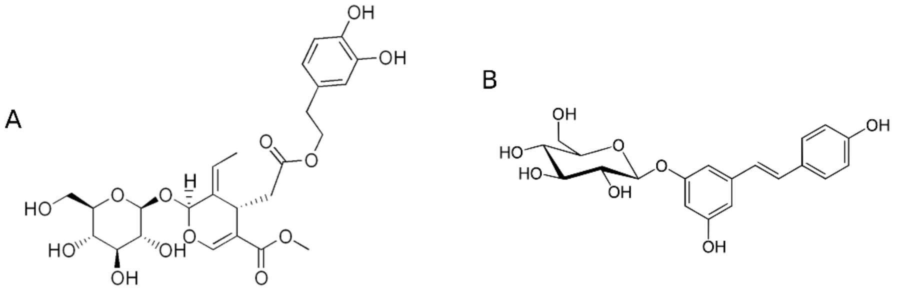

Figure S1. Oleuropein (**A**) and polydatin structures (**B**).

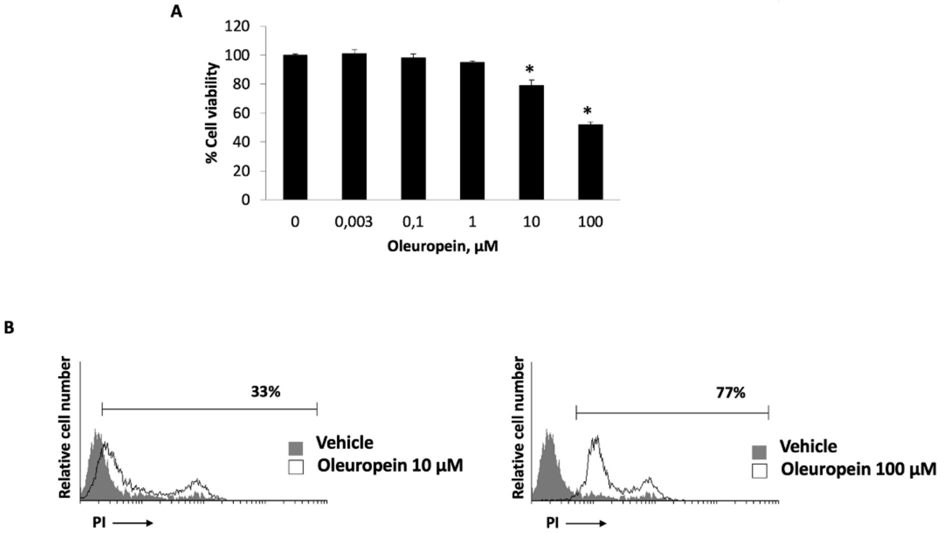

Figure S2. Oleuropein effects on huOB cell viability (**A**) Cell viability in huOB cells treated for 24 h with oleuropein. Data are the mean + SD of three experiments. * $p < 0.05$ vs. untreated cells. (**B**) PI incorporation was analyzed by flow cytometry in huOB cells treated with oleuropein 10 and 100 µM. Histograms are representative of one of three separate experiments. Percentages represent PI positive cells.

Table S1. List of primers used in ddPCR assay.

Target Name	Probe Fluorophore	ID
**ACTB**	FAM	dHsaCPE5190199
**ALP**	HEX	dHsaCPE5039245
**BGLAP**	FAM	dHsaCPE5031314
**CKM**	HEX	dHsaCPE5057839
**COL1A1**	FAM	dHsaCPE5034390
**DSPP**	FAM	dHsaCPE5026308
**GAPDH**	FAM	dHsaCPE5031596
**MYF5**	FAM	dHsaCPE5026294
**MYOG**	HEX	dHsaCPE5035593
**VDR**	HEX	dHsaCPE5058215
